# Genome Characterization, Prevalence and Distribution of a Macula-Like Virus from *Apis mellifera* and *Varroa destructor*

**DOI:** 10.3390/v7072789

**Published:** 2015-07-06

**Authors:** Joachim R. de Miranda, R. Scott Cornman, Jay D. Evans, Emilia Semberg, Nizar Haddad, Peter Neumann, Laurent Gauthier

**Affiliations:** 1 Department of Entomology, Pennsylvania State University, State College, PA 16802, USA; 2 School of Biological Sciences, Queen’s University Belfast, Belfast BT9 7BL, UK; 3 Department of Ecology, Swedish University of Agricultural Sciences, Uppsala 750 07, Sweden; E-Mail: emilia.semberg@slu.se; 4 Bee Research Laboratory, US Department of Agriculture, Beltsville, MD 20705, USA; E-Mails: scott.cornman@gmail.com (R.S.C.); jay.evans@ars.usda.gov (J.E.); 5 Bee Research Department, National Center for Agricultural Research and Extension, Baqa’ 19381, Jordan; E-Mail: drnizarh@gmail.com; 6 Institute of Bee Health, Vetsuisse Faculty, University of Bern, Bern CH-3001, Switzerland; E-Mail: peter.neumann@vetsuisse.unibe.ch; 7 Agroscope, Bee Research Center, Schwarzenburgstrasse 161, Bern CH-3003, Switzerland; E-Mail: laurent.gauthier@agroscope.admin.ch

**Keywords:** honeybee, *Apis mellifera*, *Varroa destructor*, virus, *Tymoviridae*, Maculavirus, Marafivirus, Tymovirus

## Abstract

Around 14 distinct virus species-complexes have been detected in honeybees, each with one or more strains or sub-species. Here we present the initial characterization of an entirely new virus species-complex discovered in honeybee (*Apis mellifera* L.) and varroa mite (*Varroa destructor*) samples from Europe and the USA. The virus has a naturally poly-adenylated RNA genome of about 6500 nucleotides with a genome organization and sequence similar to the Tymoviridae (*Tymovirales; Tymoviridae*), a predominantly plant-infecting virus family. Literature and laboratory analyses indicated that the virus had not previously been described. The virus is very common in French apiaries, mirroring the results from an extensive Belgian survey, but could not be detected in equally-extensive Swedish and Norwegian bee disease surveys. The virus appears to be closely linked to varroa, with the highest prevalence found in varroa samples and a clear seasonal distribution peaking in autumn, coinciding with the natural varroa population development. Sub-genomic RNA analyses show that bees are definite hosts, while varroa is a possible host and likely vector. The tentative name of Bee Macula-like virus (BeeMLV) is therefore proposed. A second, distantly related *Tymoviridae*-like virus was also discovered in varroa transcriptomes, tentatively named Varroa Tymo-like virus (VTLV).

## 1. Introduction

Honeybees (*Apis mellifera* L.) are hosts to a large number of viruses [1,2]. Most were discovered and characterized serologically during the 1970s and 1980s at Rothamsted Research International and many have recently been sequenced [2]. Although as many as 26 virus-like entities have been described, many of these are closely related by serology [1,3] or nucleotide identity [4,5] and a more accurate description is one of 14 distinct honeybee virus species-complexes, each with a spectrum of closely related, but unique viruses [2,4,5]. Here, we describe the discovery, genomic characterization, and distribution of a new virus species-complex, found naturally in honeybees and its parasitic ectoparasite, *Varroa destructor*, related to Macula-, Marafi- and Tymoviruses (*Tymovirales*; *Tymoviridae*).

## 2. Materials and Methods

### 2.1. Sample Origins, RNA Purification and cDNA Synthesis

The origin of the field samples, the RNA purification and the cDNA synthesis protocols are described in original publications for the 2000 Pennsylvania sample [6], the 2002 French survey samples [7,8], the 2007 Jordanian samples [9], and the 2008 USA varroa samples [10,11,12]. Virus samples for cloudy wing virus (CWV), Bee virus X (BVX), Arkansas bee virus (ABV), and Berkeley bee picorna-like virus (BBPV) were collected or propagated by standard methods [1,2]. The presence of the indicated virus was verified by ELISA [2] using reference antisera obtained from Rothamsted Research International. Total RNA was extracted from the various reference virus samples using RNeasy^®^ columns (Qiagen, Hilden, Germany). Approximately 1 μg RNA was converted to cDNA with the GoScript cDNA synthesis kit (Promega, Madison, WI, USA) using random hexamer primers, diluted 10-fold with water, and was stored at −20 °C until further use.

### 2.2. PCR and qPCR Assays

**Figure 1:**
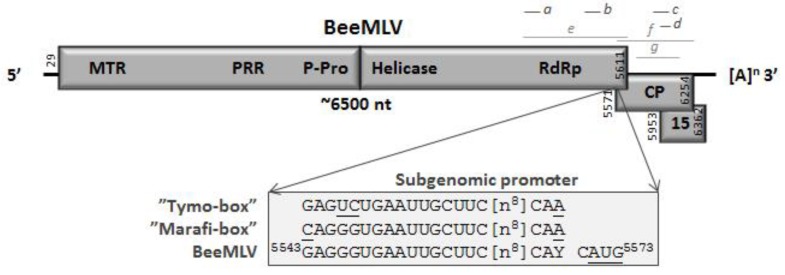
Genome organization of BeeMLV. The estimated genome size is indicated as are the presence of a natural 3′ poly-A tail and the location of overlapping open reading frames coding for the capsid protein (CP), an unknown 15 kD protein (P15) and the large polyprotein containing the domains MTR (methyl transferase), PRR (Proline-rich region), P-Pro (endo-peptidase), Helic (NTPase/helicase) and RdRp (RNA-dependent RNA polymerase), separated by the location of the putative P-Pro cleavage site in between the P-Pro and Helic domains. Additionally indicated are the location and sequence of the putative BeeMLV sub-genomic RNA promoter, compared to its homologues for the Tymo- and Marifiviruses. The lines above the genome map identify the locations of the nucleotide (*a*) and RdRp (*b*) plus CP (*c*) amino acid sequences used in the phylogenetic analyses (light grey) and of the quantitative RT-qPCR assays for either genomic (*d*, *e*) or sub-genomic + genomic (*f*, *g*) virus RNA quantification (dark grey).

For qualitative PCR the cDNA samples were amplified using the GoTaq system (Promega) in 20 μL reaction volumes containing 10 μL of a 1/10 dilution of random-primed cDNA [7], 0.4 μM of each primer (Supplementary Table S1) and a cycling profile consisting of 2 min at 95 °C followed by 35 cycles of 30 s at 95 °C: 30 s at 55 °C: 30 s at 72 °C followed by 10 min at 72 °C. Quantitative PCR was performed using the Eva-Green system (Bio-Rad, Hercules, CA, USA); 20 μL reaction volumes with 2 μL of the 1/10 diluted cDNA template [7], 0.2 μM of each primer and a cycling profile of 30 s at 95 °C plus 35× (10 s at 95 °C: 10 s at 56 °C: fluorescence read) followed by a Melting Curve (MC) analysis at 5 s/0.5 °C intervals from 50 °C to 95 °C to distinguish true target amplifications from PCR artifacts. The primers used for the quantification of sub-genomic and/or genomic viral RNA (Figure 1; fragments “*a*”, “*b*”, “*c*”, “*d*”) are given in Supplementary Table S1, as well as those for the quantification of Tobacco Mosaic Virus (TMV), which was included as a passive reference during the preparation of the French 2002 survey samples [8,13]. All assays were run in duplicate for each sample. The mean C_q_ values for the samples were converted to absolute copy numbers using a calibration curve derived from the linear regression of C_q_ value onto Log_10_[template] for a 10-fold dilution series of an external standard of known concentration. The regression slopes were also used to determine the reaction efficiencies of the different assays (Supplementary Table S1; [14]). A constant of one copy was added to the value of each reaction to permit instances of non-detection (zero values) to be log-transformed [15], thus allowing virus-free samples to be included in parametric statistical analyses (see later). The copy numbers per reaction were then multiplied by the various dilution factors [8] to calculate the estimated copy number of virus per bee or mite. The TMV data was then used to adjust the virus data for sample-specific differences in the quantity and quality of RNA [16].

### 2.3. Cloning, Sequencing, and Assembly

The sequence of the European variant of bee Macula-like virus (BeeMLV^EU^) was assembled from Illumina sequence data derived from RNA of 100 varroa mites from 10 colonies of apiary #878 [7,8], and was produced, processed and assembled by Fasteris Life Science Co. (Geneva, Switzerland). The RNA for this was prepared by phenol-chloroform extraction followed by ethanol precipitation, and was enriched for mRNA by affinity purification on oligo-dT columns. Around 25 million 100 nt paired reads were quality-filtered; adapter sequences were removed and the resulting data was assembled at a range of kmer values using the VELVET [17] and OASES programs [18] made available on-line by the European Bioinformatics Institute (www.ebi.ac.uk). The 823 BeeMLV contigs (median length of N50 = 520 bp) were scaffolded using the SeqMan program (Lasergene 7.0; Dnastar Inc., Madison, WI, USA) and finally edited manually. The assembled Illumina sequence was confirmed by chain-termination sequencing [19] of cloned and uncloned RT-PCR fragments amplified from varroa and bee samples from apiaries #28 and #878 of the 2002 French survey [7,8], covering 99% of the assembled genome (Supplementary Figure S1). The 3' terminus was obtained by priming the natural poly-A tail of the virus with anchored oligo-dT (V[T]^16^) for cDNA synthesis, followed by RT-PCR with a virus-specific forward primer. The sequences of the USA variant of bee Macula-like virus (BeeMLV^USA^) and of varroa Tymo-like virus (VTLV) were assembled from 454 and Illumina sequence data available from Trinity, a composite varroa transcriptome database (Genbank accession pending) of varroa samples with a variety of biological and geographic origins. The BeeMLV 5′ terminus was approximated by the consensus of the EU and USA assemblies.

### 2.4. Phylogenetic and Variability Analyses

The phylogenetic position of BeeMLV and VTLV within the Tymoviridae was determined by aligning the RNA-dependent RNA polymerase (RdRp) and capsid protein (CP) amino acid sequences (Figure 1; fragments “*e*” and “*f*”) to those of other Macula-, Marafi- and Tymoviruses (Supplementary Table S2) using CLUSTAL-Omega [20,21] and inferring the evolutionary relationships using Minimum Evolution criteria as implemented by MEGA6 [22]. All positions containing gaps were excluded from the analysis resulting in 301 (RdRp) and 167 (CP) characters for analysis. The evolutionary distances were computed using the Poisson correction method [23] and are given as amino acid substitutions per site. The relationships between different biological and geographical BeeMLV isolates was determined from a 427 nucleotide section of the capsid protein region (Figure 1; fragment “*g*”) as described above, except that the evolutionary distances were computed using the Maximum Composite Likelihood method [22]. Bootstrap analysis (500 replicates) was used to determine the statistical strength of the partitions. The variability estimates were determined from the pairwise *p*-values calculated by MEGA6 from CLUSTAL-Omega alignments of the eight BeeMLV and two VTLV assemblies. All ambiguous positions were removed for each sequence pair.

### 2.5. Statistical Analyses

The non-random association of the prevalence of BeeMLV in different tissues, or across seasons, was tested using contingency-χ^2^ analyses. The BeeMLV titers were logarithmically-distributed and were therefore log-transformed prior to parametric analyses. Differences between means were tested using *t*-tests and quantitative associations between groups of samples were tested by correlation analyses [15].

## 3. Results

### 3.1. Discovery

The origin of the present discovery was two clones, obtained inadvertently during the cloning and sequencing of deformed wing virus (DWV) in Pennsylvania in 2001 [6]. The sequences of these clones were distantly-related to the RNA-dependent RNA polymerase (RdRp) and capsid protein (CP) of Tymoviruses, a group of viruses infecting plants and transmitted by phloem-sucking insects [24,25], and were, therefore, initially thought to belong to a contaminating plant virus. However, in 2005 the genome sequence of a macula-like virus infecting the silkmoth *Bombyx mori* was published [26]. Subsequently, another insect-based tymoviridae-like virus was described [27]. Finally, numerous sequence reads related to the original clones were recovered from several *Varroa destructor* transcriptome datasets [10]. Since *Varroa destructor* is an obligate parasite that only feeds on bee haemolymph [28], and has no contact at all with plants, the possibility therefore arose that these “contaminant” sequences could, in fact, represent a true virus of honeybees or their parasites.

### 3.2. Genome Sequencing and Analysis

The first task was to assemble the complete genome sequence, which was achieved using a combination of chain termination [19] and Next Generation (454, Illumina) sequencing of samples from the USA and France. The BeeMLV genome is around 6500 nucleotides long, similar to that *Bombyx mori* Macula-like latent virus (BmMLV) and Culex Tymo-like virus (CuTLV), and has three overlapping open reading frames (ORFs). The first ORF encodes a large polyprotein containing several non-structural proteins, such as a methyl transferase (MTR), a proline-rich region (PRR), believed to act as a hinge between the MTR and P-Pro domains [29], a papain-like endo-peptidase (P-Pro [30]; an NTPase/helicase domain (Helic) and an RNA-dependent RNA polymerase (RdRp). The endo-peptidase in particular is highly characteristic of the *Tymoviridae*, cleaving the large ORF1 polyprotein into an N-terminal protein containing the MTR, PRR and P-Pro functions and a C-terminal protein containing the replicative proteins [29,30]. The second ORF encodes a ~24 kDa capsid protein and the third ORF a 14~15 kDa protein of unknown function (Figure 1) with clear homologues in the BmMLVand CuTLV genomes [26,27].

Many of the plant Tymoviridae also have an additional, large ORF in the 5′ end of the genome coding for a movement protein (MP [29]; Supplementary Table S2), a common protein in plant RNA viruses specifically involved in cell-to-cell virus transmission through plasmodesmata [31], as well as in pathology and suppression of the host RNAi antiviral defence mechanism [32]. Such a protein would have a less obvious role in purely insect viruses, except for suppressing host defences (although other proteins can also fulfill this role), and its absence from the BmMLV, CuTLV and BeeMLV genomes is therefore not unusual.

The BeeMLV genome is naturally polyadenylated, which made it possible to identify the 3′ terminus precisely. Both Macula- and Marafiviruses also have poly-A tails [24,25], while the Tymoviruses have instead a tRNA-like structure for stabilizing the 3′ end ([24]). The 3′ untranslated region is rather short (49 nt) compared to those of other members of the Tymoviridae (usually ~150 nt), unless this is calculated from the stop codon of the CP ORF (158 nt). It is, of course, entirely possible that the 3′ terminal region has multiple functions, combining its usual role in genome replication with the presence of a functional P15 ORF.

A highly conserved sub-genomic RNA promoter region (“Tymo-box” or “Marafi-box” [33,34,35]) was identified immediately prior to the CP ORF (Figure 1). This region is characterized by a highly conserved stretch of 16 nucleotides, eight nucleotides upstream of the putative start of transcription, characterized by another conserved section of three nucleotides. The putative BeeMLV sub-genomic promoter “box” differs across these two regions by only two nucleotides from the “Marafi-box” and by three nucleotides from the “Tymo-box” (Figure 1), with all variants occurring in positions where this is also tolerated elsewhere among the Tymoviruses and Marafiviruses [34]. The only unusual aspect is the slight difference between the European and USA variants of BeeMLV, with either a “CAT” (Europe) or a “CAC” (USA), signaling the putative three-nucleotide start of the sub-genomic RNA. Neither the Maculaviruses (including BmMLV) nor the unassigned CuTLV have a typical Tymo-box or Marafi-box [25,26,27,34], although they may, of course, have alternative means for generating sub-genomic RNAs for translating the 3′ ORFs. A comparison of the main genomic features of BeeMLV with those of the Tymo-, Marafi- and Maculaviruses is found in Supplementary Table S2.

A number of varroa and honeybee transcriptome sequence libraries from the USA were screened for BeeMLV sequences. The virus was not found in honeybee transcriptomes from an extensive survey of colonies affected, or not, by Colony Collapse Disorder (~60 million reads combined; [11]), and only a few dozen reads were recovered from another set of bee larval transcriptomes from the University of Georgia (~57 million reads combined; [12]). However, varroa transcriptomes are a different story. BeeMLV accounted for ~4% of reads from a pool of three 454 varroa transcriptome libraries (~1.5 million reads combined), with most of the hits coming from the varroa sample from Penn State University and for ~0.015% of reads in two Illumina varroa transcriptomes (~45 million reads combined; [12]). These searches also recovered a second, distantly-related Tymoviridae-like virus (tentatively named varroa Tymo-like virus; VTLV) in the Penn State University varroa sample, accounting for ~2% of the 454 reads, enough to assemble a rough draft of the genome and for phylogenetic analyses. The sequences reported here have been submitted to GenBank under accession numbers KT162924 (BeeMLV^EU^), KT162925 (BeeMLV^USA^) and KT162926 (VTLV).

**Figure 2:**
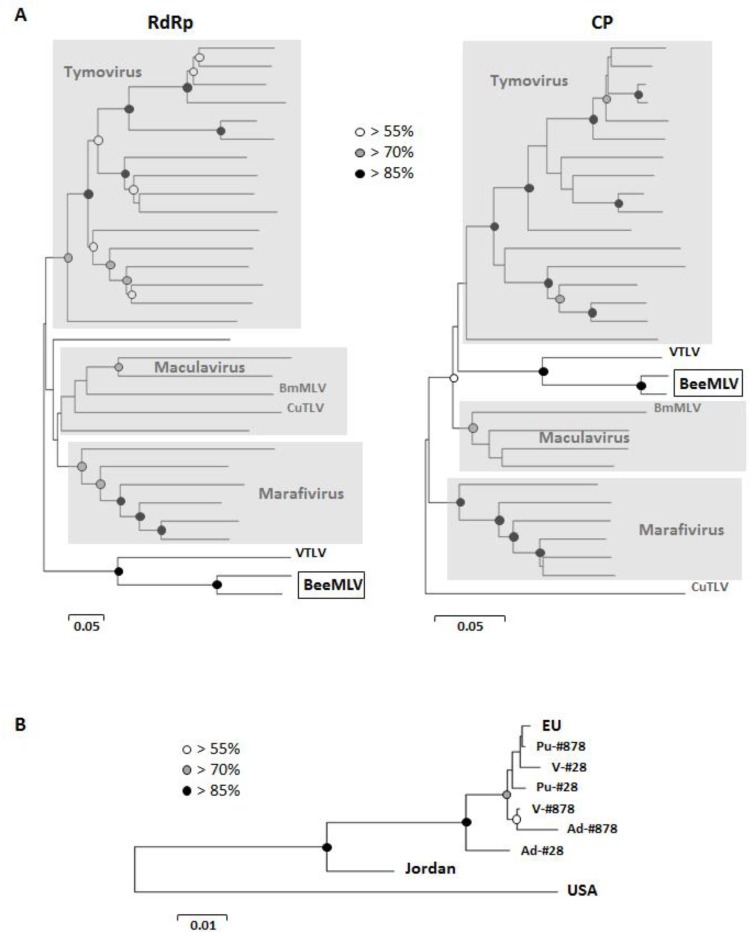
(**A**) Unrooted Minimum Evolution phylograms describing the relationship between BeeMLV and other viruses in the *Tymoviridae,* based on the RNA-dependent RNA polymerase and capsid protein amino acid sequences. The scale bar represents the inferred evolutionary distance in amino acid substitutions per site. Bootstrap support for the various nodes is indicated by the white (>55%), grey (>70%) and black (>85%) circles. The Tymovirus, Marafivirus and Maculavirus genera are marked with grey-shaded areas. The two insect-infecting *Tymoviridae*; *Bombyx mori* Macula-like latent virus (BmMLV) and Culex Tymoviridae-like virus (CuTLV) are indicated in both phylograms, as well as a second Tymoviridae-like virus (VTLV) recovered in these studies; (**B**) Unrooted Minimum Evolution phylogram describing the relationship between different geographic and biological BeeMLV isolates, based on the capsid protein nucleotide sequence. The scale bar represents the inferred evolutionary distance in nucleotide substitutions per site. Bootstrap support is indicated as for Figure 2A. The taxa are consensus nucleotide sequences from Europe, Jordan, the USA and the pupal (Pu), adult (Ad) and varroa (V) pooled samples from apiaries in northern France (#28) and southern France (#878).

### 3.3. Phylogenetic Analyses

The phylogenetic analyses of the RdRp and CP regions position BeeMLV firmly within the *Tymoviridae*, but outside the Tymovirus, Marifivirus and Maculavirus genera (Figure 2A), with as closest relative the second Tymoviridae-like virus recovered from the varroa 454 and Illumina transcriptome data-sets (VTLV), with perfect bootstrap support for their internal separation and their combined separation from the remaining Tymoviridae. BeeMLV^EU^, BeeMLV^USA^ and VTLV are each internally >92% identical at the nucleotide level, based on the variation between independent assemblies, which translates to >91% and >96% identity at amino acid level for the main polyprotein and the CP respectively (Supplementary Table S4). The two BeeMLV strains are >70% identical at nucleotide level (>72% and >80% for the polyprotein and CP amino acid sequences) while BeeMLV and VTLV are clearly very distinct, with only about 50% nucleotide identity (48% and 40%, respectively, for the polyprotein and CP amino acid sequences). There is no phylogenetic distinction between the BeeMLV nucleotide sequences derived from honeybee or varroa-mite samples (Figure 2B) and placement of the Jordanian isolate implies a broad, geographically driven variability for this virus covering the sequence space between the European and USA isolates, that these isolates are best regarded as variants of a single virus species, rather than separate species.

### 3.4. Reference Collection Analysis

The next question was whether this virus had been described previously, as part of the ground-breaking work of Lesley Bailey and Brenda Ball at Rothamsted Research in England, between 1956 and 2006. Of all the honeybee RNA viruses described by Bailey and Ball, there are four left that are serologically unique, unrelated to already sequenced viruses and that have not yet bee sequenced. These are cloudy wing virus (CWV), bee viruses X and Y (BVX; BVY), Arkansas bee virus (ABV) and Berkeley bee picorna-like virus (BBPV). However, much is known about the physical, chemical, and biological properties of these viruses (Supplementary Table S3; [1]). All form icosahedral particles, but those of CWV are much smaller (~17 nm) than those of the Tymoviridae (~30 nm), while BVX and BVY have slightly bigger particles (~35 nm). The capsid proteins of BVX and BVY (50~52 kDa), ABV (~43 kDa) and BBPV (32, 35 and 37 kDa) are far larger and/or more numerous than the predicted BeeMLV capsid protein (~24 kDa). Only the CWV capsid protein (~19 kDa) comes close to the size of the BeeMLV capsid protein. However, the CWV RNA genome is much smaller (~1700 nt) than the ~6500 nt BeeMLV genome, while the BBPV genome is much larger, at ~9000 nt. Since BeeMLV could, furthermore, also not be detected by RT-PCR in any of the reference virus RNA samples (data not shown), the most parsimonious conclusion is that this virus has not previously been described in honeybees.

### 3.5. Prevalence and Distribution

**Figure 3:**
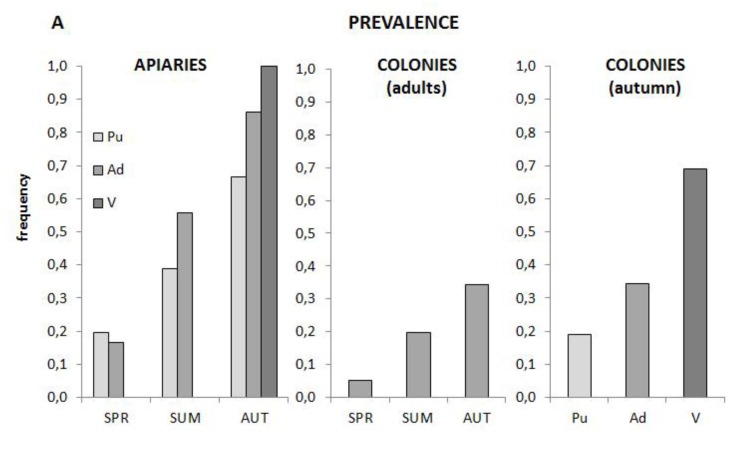

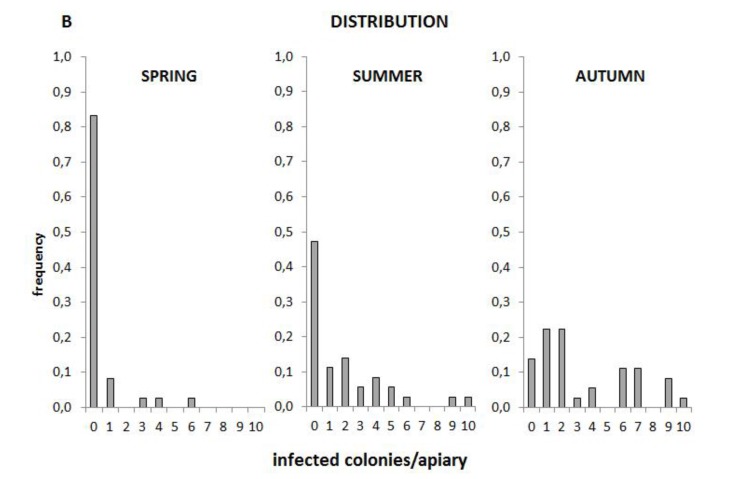
(**A**) Prevalence of BeeMLV in *A. mellifera* pupae (light grey), adults (medium grey) and *V. destructor* (dark grey) collected during Spring, Summer and Autumn of 2002 from 360 colonies in 36 French apiaries. The data are analyzed at the apiary level, and at the individual colony level for all seasons (adult samples only) and for all three sample types (autumn samples only); (**B**) Distribution of the number of infected colonies per apiary throughout the season, as determined from the adult colony samples.

A screen of cDNA samples from the 2002 French honeybee virus survey of 360 colonies in 36 apiaries throughout France [7,8] showed that BeeMLV was both common and abundant in bee colonies, particularly in the varroa mite samples. The prevalence increases from spring through summer to autumn with the greatest prevalence in mites, followed by adults and pupae, both when analyzed at the apiary level and the individual colony level (Figure 3A). The distribution of this prevalence among the 10 colonies in each apiary develops a slightly bimodal character as the season progresses (Figure 3B), with either few or many colonies in an apiary infected, suggesting that local factors may influence prevalence. The prevalence data was positively associated across seasons (χ^2^_(3)_ = 26.94; *P* < 0.005), *i.e.*, colonies that were positive in spring, or became positive during summer, were more likely than not also positive in autumn. Among the autumn samples there was also positive association between the prevalence in adults and pupae (χ^2^_(1)_ = 20.24; *P* < 0.005) but not between either adults or pupae and mites. Both the seasonal and adult-pupae associations are less clear for the pooled apiary samples, although this may simply be a statistical consequence of having only 1/10^th^ the amount of data to analyze.

An independent extensive virus survey found that BeeMLV was also common and abundant in Belgian honeybee colonies [36]. However, BeeMLV has not so far been detected in in a number of extensive geographic surveys of Swedish and Norwegian honeybee colonies from varroa-infested and non-infested regions (~400 samples from throughout Norway and Sweden; unpublished data), nor in preliminary virus screens of numerous heavily varroa-infested experimental colonies in Sweden [37,38], nor in samples from Isle d’Ouessant, a varroa-free territory off the west coast off France [7,8]. More pertinently, BeeMLV was also not detected in a large collection of nearly 100 failing, virgin, and newly-mated French honeybee queens [39], collected from a similar geographic range as the 2002 French survey samples. As mentioned earlier, in the USA BeeMLV was detected abundantly in a few varroa transcriptomes, but rarely in (unrelated) sets of honeybee transcriptomes from a much wider geographic range of samples.

### 3.6. Genomic and Sub-Genomic BeeMLV Titers

**Figure 4:**
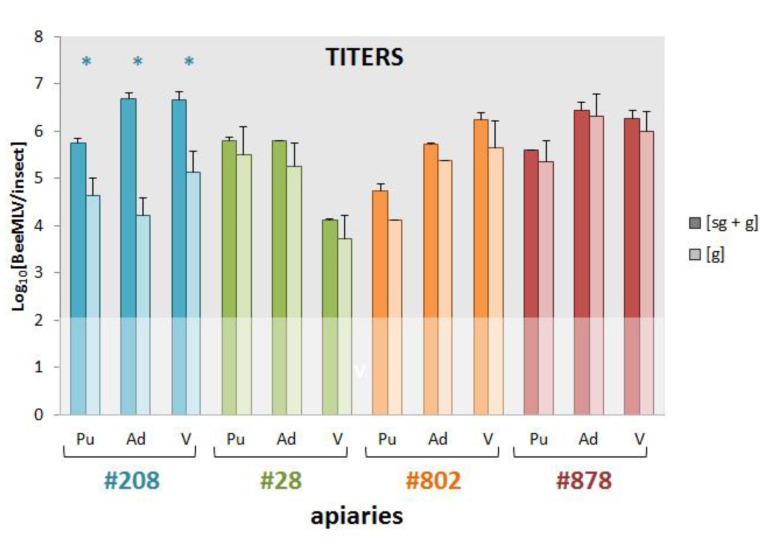
Log_10_[BeeMLV] titers of genomic (g) and sub-genomic plus genomic (sg + g) BeeMLV RNA in pupae (Pu), adults (Ad) and varroa (V) samples collected in Autumn 2002 from four apiaries in Western (#208; blue), Northern (#28; green) Eastern (#802; orange) and Southern (#878; red) France. Values represent the mean and standard deviation of two independent assays for only genomic RNA, and two assays for genomic-plus-subgenomic RNA (located 5′ and 3′ respectively of the putative start of the sub-genomic RNA; Figure 1), run on the pooled cDNA from 10 colonies in each apiary, for each of the sample types. The faded area represents the limit of detection. Significant differences between the genomic and genomic-plus-subgenomic titers, as determined by *t*-tests, are marked by an asterisk (*).

The next question to address was whether bees and mites are true infection hosts of the virus, or merely carriers of passively acquired virus particles. The presence of a sub-genomic RNA, for expression of the second ORF, makes it possible to differentiate active and passive states, since only actively replicating virus produces sub-genomic RNA. We designed two separate qPCR assays in the RdRp region, 5′ of the putative start of the sub-genomic RNA (Figure 1) and two qPCR assays in the sub-genomic region. If there is active production of large amounts of sub-genomic RNA, then this would be shown by quantitative differences between the two sets of assays. The assays were run on cDNA samples from pupae, adult bees, and varroa mites from four apiaries, located in Northern, Eastern, Southern and Western France, with each cDNA sample a pool of individual cDNAs from the 10 colonies in each apiary [7,8]. The results are shown in Figure 4. For all samples there is more BeeMLV RNA in the sub-genomic region, where both genomic and sub-genomic RNA are available, than in the region where only genomic RNA is available. For the samples from apiary #208 these differences are large (several orders of magnitude) and highly significant for all sample types. For the other apiaries the differences are smaller, and less significant. The highest titers are generally found in adult bees and mites, except for apiary #28 where the mite titers were significantly lower than those of the adults and pupae. These data confirm a number of things. First, that BeeMLV does produce sub-genomic RNA, as proven most emphatically by the samples from apiary #208. Second, the lack of large sub-genomic excess in apiaries #28, #802 and #878 (most likely because the virus is in a different stage of its infection) emphasizes the significance of the results from apiary #208, and suggests that the assays employed accurately reflect the quantitative differences between the two genomic regions. Third, honeybees, particularly the adult bees, are true infectious hosts of BeeMLV. The fact that all three sample types from apiary #208 show a similarly large excess of sub-genomic RNA is particularly illustrative, since it confirms the replication of the virus throughout the colony. The host status of the mites is less certain, since these were recovered from adult bees [7,8] and their signal may simply reflect their latest blood-meal. A significant observation here is that, in those samples where there was little evidence of sub-genomic RNA in bees (apiaries #28, #802 and #878), there was also no sub-genomic RNA in the mites. When there was sub-genomic RNA in the bee samples (apiary #208), it was also found in the corresponding mite samples. However, lack of replication in bees should not prevent the mite (if it is a true host) from replicating the virus. This suggests that the virus signal in the mites follows primarily that of the bees, through feeding. Even if there is true replication in mites, the signal may well be sub-merged by the viral RNA (without signal of replication) acquired from the bee. Ultimately, the host status of the mite can only be established by looking at infection in mite tissues proper, without contaminating influences from bee-derived nucleic acids.

## 4. Discussion

The genome size, organization, predicted functional protein domains, polyprotein processing, sub-genomic RNA promoter and phylogenetic analyses clearly identify BeeMLV as a member the *Tymoviridae* [24]. It appears closest overall to the Maculavirus genus, [25] but with a number of idiosyncrasies and affinities to the other two genera, such that it is probably best classified as an unassigned member of the *Tymoviridae*, with its genus status to be resolved when similar insect-specific Tymo-like viruses are reported. The phylogenetic relationships between the three genera are in any case rather indistinct, with a few other assigned and unassigned species floating in between the groups, attesting to a considerable degree of homoplasy in the data (possibly from horizontal transfer of genetic material between viruses; a common disturbing factor underlying difficult virus phylogenetic reconstructions), and perhaps also to a degree of flexibility in genome organization, replication and translation strategies (Supplementary Table S2) which are other important virus classification criteria.

The other main point of discussion is the origin and primary host of the virus, which ideally should be reflected in its name. There are a number of indirect indicators that this virus may be more closely linked to *Varroa destructor* than to *Apis mellifera* (elevated prevalence and titers in varroa samples, seasonal patterns, over-representation in varroa *vs* honeybee transcriptomes), but none of these are by themselves conclusive. Establishing host status is easier for bees than for mites, as illustrated by the sub-genomic analyses. Varroa mites are intimately connected with honeybees, feeding almost continually on the bee haemolymph; in many ways little more than a small bladder of honeybee blood, with all the honeybee viruses that would contain. Nucleic acids extracted from varroa contain significant levels of honeybee-derived nucleic acids [10]. The presence in varroa RNA of honeybee viruses, or even transient virus replication or translation RNA intermediates, does not in itself make varroa a virus host. Conversely, the detection of BeeMLV in honeybee pupal and adult RNA samples, or transcriptomes, also does not necessarily identify honeybees as a host. These samples may well have contained varroa mites that, as a possible true host in this scenario, could have contributed BeeMLV transcripts to the “honeybee” RNA being analyzed. However, the level of contamination would be considerably less in this case, and probably insufficient to explain the considerable BeeMLV titers in both the adult and pupal samples from the French survey, and certainly insufficient to explain the high excess of sub-genomic BeeMLV RNA in the bee samples from apiary #208. Bees are definitely a host. Whether mites are also a true host will require other types of evidence, such as (electron) microscopy of mite tissues, which are less affected by the contamination of mite tissues with honeybee-derived nucleic acids. Whether honeybees are also the original host of BeeMLV is a more difficult, and in the current context, probably a less relevant question. At one point we wondered whether the different sequence variants found represented either honeybee- or varroa-adapted BeeMLV strains, but the phylogenetic analyses do not support this. The stark contrast between the high abundance of BeeMLV in France (this study), Belgium [36] and the USA varroa transcriptomes with its absence from the pan-USA honeybee transcriptomes [11], Scandinavia and the French honeybee queen study [39], all extensive, wide-ranging surveys from regions with long histories of varroa infestation, is also puzzling. It suggests an incidental virus, rather than an endemic one, capable of replicating in bees and, thus, accumulating in mites, and which is possibly transmitted by mites to stimulate regional dispersal and local epidemics, but whose origins and initial establishment remain a mystery. These questions require further investigation. Other viruses closely linked to varroa but that are endemic to bees, such as acute bee paralysis virus and relatives [4], and deformed wing virus [5], tend to be more evenly co-distributed with varroa. Honeybee queens are almost never infested with varroa mites either as pupae or adults [40,41] but can still be infected with deformed wing virus through oral and sexual transmission [5,42,43] such that virus, even if initially undetectable, is inevitably available for proliferation when mites are first introduced to the system [44,45], stabilizing the co-distribution. The distribution of BeeMLV does not fit this pattern.

For these reasons we propose the generic name of Bee macula-like virus (BeeMLV), rather than a host-specific name, reflecting its replication in honeybees and close association with varroa mites, its uncertain origins, and its taxonomic affiliation.

## Supplementary Files

Supplementary File 1
