# Supplementary material for: Genome Characterization, Prevalence and Distribution of a Macula-Like Virus from Apis mellifera and Varroa destructor
Source: Viruses. 2015 Jul 6;7(7):3586–602. doi: 10.3390/v7072789 (PMC4517118; doi:10.3390/v7072789)
Supplement: Supplementary File 1 [file viruses-07-02789-s001.pdf]

Supplementary Information

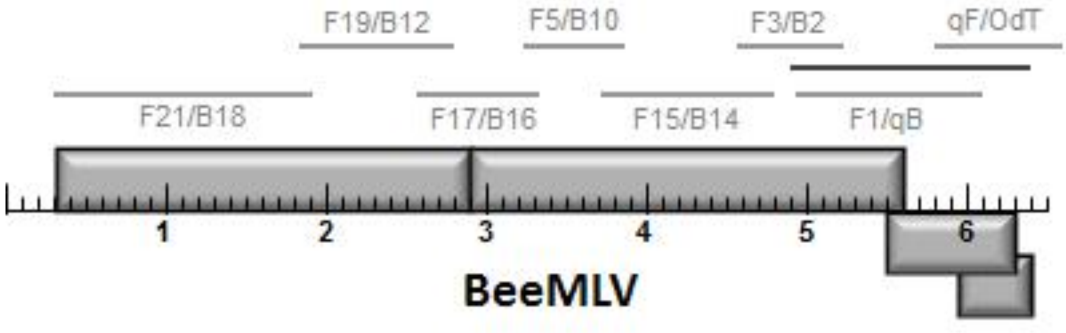

Figure S1. Sequencing map. Map of PCR fragments (light grey) used for confirmatory "Sanger" sequencing of the European BeeMLV genome, and the location of the original USA partial clone (dark grey).

Table S1. Primers. Name, sequence and genome location of primers for amplifying RT-PCR fragments for sequence analyses (unshaded) and quantitative RT-qPCR analyses (shaded grey). Also shown are the fragment sizes, amplification efficiency (*E*), log-linearity between *Cq* value and template amount over 8 orders of magnitude (r^2) and melting temperatures (T_m) of the PCR products [14].

Assay	Primers	Sequence (5′-3′)	Size	Location 3′	*E*	r^2	T_m
Phylogeny	BeeMLV-F	ATCCCTTTTCAGTTCGCT	438	5807	*n.a.*	*n.a.*	*n.a.*
	BeeMLV-R	AGAAGAGACTTCAAGGAC		6210			
Sequence	BeeMLV-qF	GCTCGTATTCCTGCTACTCC	707	5745	*n.a.*	*n.a.*	*n.a.*
	Oligo-dT	TTTTTTTTTTTTTTTTTTTTTTTTTV		6408			
	BeeMLV-F1	CTTCGTCAGCGGAGACGACTC	853	5046	*n.a.*	*n.a.*	*n.a.*
	BeeMLV-qB	TTGACGGATTGAGGGATGC		5860			
	BeeMLV-F3	ACCGTTCCGACCCGGACTG	636	4515	*n.a.*	*n.a.*	*n.a.*
	BeeMLV-B2	GATCGATCTGTATTTTGAAGCG		5111			
	BeeMLV-F15	TACCACGCCGTCACATGGG	1088	3557	*n.a.*	*n.a.*	*n.a.*
	BeeMLV-B14	CACGTAATCATGCATGAG		4609			
	BeeMLV-F5	ACCAATTTACGACCTGAGTG	581	3099	*n.a.*	*n.a.*	*n.a.*
	BeeMLV-B10	CGAACGAGTCAGCGCCAC		3643			
	BeeMLV-F17	TGCCCGACTGCCAGCTTG	664	2522	*n.a.*	*n.a.*	*n.a.*
	BeeMLV-B16	TTGGTGAGCGCGGTTTCCCA		3149			
	BeeMLV-F19	TCTCTTCCGCCGTTTCGG	1047	1548	*n.a.*	*n.a.*	*n.a.*
	BeeMLV-B12	AGCCAGTGCGGAAAGATG		2560			
	BeeMLV-F21	GCTCAGCGCCAGCATCCACC	1490	0080	*n.a.*	*n.a.*	*n.a.*
	BeeMLV-B18	GGGCCGAAACGGCGGAA		1534			

Table S1. *Cont.*

Assay	Primers	Sequence (5′-3′)	Size	Location 3′	*E*	r²	T_m
RT-qPCR	BeeMLV-852F<br>BeeMLV-949R	CAAAGGCATCGACTACGTCTTCT<br>CGAGCACGGCCTCAAGAG	98	sub-genomic	1.945	0.999	84.0 ºC
	BeeMLV-F<br>BeeMLV-949R	ATCCCTTTTCAGTTCGCT<br>CGAGCACGGCCTCAAGAG	142	sub-genomic	1.988	0.999	84.8 ºC
	BeeMLV-F1<br>BeeMLV-B2	CTTCGTCAGCGGAGACGACTC<br>GATCTGTATTTTGAAGCG	163	genomic	1.965	0.999	82.5 ºC
	BeeMLV-F3<br>BeeMLV-B14	ACCGCTCCGACCCCGACTG<br>CACGTAATCATGCATGAG	130	genomic	1.948	0.999	84.1 ºC
Internal Reference	Q-TMV-fwd<br>Q-TMV-rev	CATGCGAACATCAGCCAATG<br>TGTAGCGCAATGGCGTACAC	55	reference	1.995	0.999	81.5 ºC

Table S2. Viruses and accession numbers. Names, accession numbers and genomic characteristics of BeeMLV and the Tymo-Marafi- and Maculaviruses used in the phylogenetic analyses. Bold type, intensity of letter font and grey shades represent similarity with the BeeMLV genome. The genomic characteristics tabulated are (left-to-right): presence of a natural poly-A tail (Poly-A); presence of a Movement Protein (MP); presence of a Proline-rich region (PRR); presence of an endopeptidase site (P-Pro); presence of a sub-genomic RNA promoter (sg-promoter); presence of a separate ORF encoding the capsid protein (CP); presence of small overlapping ORF's at the 3′ end (3′ ORFs); presence of an intergenic region separating the main and CP ORFs (IGR).

TYMOVIRIDAE	ACRONYM	ACCESSION	GENUS	Poly-A	MP	PRR	P-Pro	Sg-promoter	CP ORF	3′ ORFs	IGR
Anagyris vein yellowing virus	AnVYV	AY751780		no	yes	**yes**	**yes**	**yes**	**yes**	no	**no**
Chayote mosaic virus	ChMV	AF195000		no	yes	**yes**	**yes**	**yes**	**yes**	no	yes
Chiltepin yellow mosaic virus	ChYMV	FN563123		no	yes	**yes**	**yes**	**yes**	**yes**	no	yes
Diascia yellow mottle virus	DiYMV	EU684141		no	yes	**yes**	**yes**	**yes**	**yes**	no	yes
Dulcamara mottle virus	DuMV	AY789137		no	yes	**yes**	**yes**	**yes**	**yes**	no	yes
Eggplant mosaic virus	EMV	J04374		no	yes	**yes**	**yes**	**yes**	**yes**	no	yes
Erysium latent virus	ErLV	AF098523		no	yes	**yes**	**yes**	**yes**	**yes**	no	**no**
Kennedya yellow mosaic virus	KeYMV	D00637		no	yes	**yes**	**yes**	**yes**	**yes**	no	yes
Mertensia leaf curl virus	MeLCV	FJ713524	Tymovirus	no	?	**yes**	**yes**	**yes**	**yes**	no	yes
Nemesia ring necrosis virus	NeRNV	AY751778		no	yes	**yes**	**yes**	**yes**	**yes**	no	yes
Okra mosaic virus	OMV	EF554577		no	yes	**yes**	**yes**	**yes**	**yes**	no	yes
Onosis yellow mosaic virus	OnYMV	J04375		no	yes	**yes**	**yes**	**yes**	**yes**	no	**no**
Physalis mottle virus	PhMV	Y16104		no	yes	**yes**	**yes**	**yes**	**yes**	no	yes
Plantago mottle virus	PlMV	AY751779		no	yes	**yes**	**yes**	**yes**	**yes**	no	yes
Poinsettia mosaic virus	PoMV	AB550789		no	yes	**yes**	**yes**	**yes**	**yes**	no	**no**
Scrophularia mottle virus	ScMV	AY751777		no	yes	**yes**	**yes**	**yes**	**yes**	no	**no**
Turnip yellow mosaic virus	TYMV	AF035403		no	yes	**yes**	**yes**	**yes**	**yes**	no	yes

Table S2. *Cont.*

TYMOVIRIDAE	ACRONYM	ACCESSION	GENUS	Poly-A	MP	PRR	P-Pro	Sg-promoter	CP ORF	3′ ORFs	IGR
Grapevine Syrah virus-1	GSyV-1	FJ436028	Marafivirus	**yes**	yes	**yes**	**yes**	**yes**	no (2)	no	**no**
Blackberry virus-S	BVS	FJ915122		**yes**	**no**	**yes**	**yes**	**yes**	no (2)	no	**no**
Citrus sudden death associated virus	CSDaV	AY884005		**yes**	**no**	**yes**	**yes**	**yes**	no (2)	**yes**	**no**
Maize rayado fino virus	MRFV	AF265566		no	yes	**yes**	**yes**	**yes**	no (2)	no	**no**
Oat blue dwarf virus	OBDV	U87832		**yes**	**no**	**yes**	**yes**	**yes**	**yes (1)**	no	**no**
Olive latent virus 3	OLV3	FJ444852		**yes**	yes	**yes**	**yes**	**yes**	**yes (1)**	**yes**	yes
Grapevine fleck virus	GFkV	AJ309022	Maculavirus	**yes**	**no**	**yes**	**yes**	no	**yes**	**yes**	yes
Grapevine red globe virus	GRGV	AF521977		**yes**	**no**	**yes**	**yes**	no	**yes**	**yes**	**no**
Fig fleck-associated virus	FFkaV	FM200426		**yes**	yes	**yes**	**yes**	no	no	no	**no**
Bombyx mori Macula-like latent virus	BmMLV	AB186123		**yes**	**no**	**yes**	**yes**	no	**yes**	**yes**	yes
Culex-originated Tymoviridae-like virus	CuTLV	JQ429443		**yes**	**no**	**yes**	**yes**	no	**yes**	**yes**	yes
Bee Macula-like virus	BeeMLV	KT162924/5	?	**yes**	**no**	**yes**	**yes**	**yes**	**yes**	**yes**	**no**

Table S3. Reference collection analysis. Comparison of the physico-chemical characteristics of BeeMLV with previously characterized and as-yet unsequenced historical viruses. Bold type, intensity of letter font and grey shading represent level of similarity.

VIRUS	SHAPE	SIZE	CAPSID PROTEINS	GENOME
Cloudy wing virus	**icosahedral**	17 nm	19 kDa	~1.4 kb
Bee virus-X	**icosahedral**	35 nm	52 kDa	?
Bee virus-Y	**icosahedral**	35 nm	50 kDa	?
Arkansas bee virus	**icosahedral**	**30 nm**	43 kDa	~5.6 kb
Berkeley bee picorna-like virus	**icosahedral**	**30 nm**	37-35-32 kDa	~9 kb
Bee Macula-like virus	**icosahedral**	**30 nm**	**24 kDa**	**~6.5 kb**

Table S4. Variability of BeeMLV and VTLV. Variability within and between the European and USA strains of BeeMLV, and between BeeMLV and VTLV, as represented by the average amino acid identities for the two main ORFs (RdRP; CP) and nucleotide identities across the genomes, as determined from comparisons between the different NGS assemblies for these viruses.

% Identity	Nucleotide	RdRp	CP
within $BeeMLV^{EU}$	0.968	0.976	0.994
within $BeeMLV^{USA}$	0.944	0.913	0.960
within VTLV	0.923	0.957	0.962
$BeeMLV^{EU}$ *vs*. $BeeMLV^{USA}$	0.708	0.723	0.809
BeeMLV *vs*. VTLV	0.519	0.488	0.404

© 2015 by the authors; licensee MDPI, Basel, Switzerland. This article is an open access article distributed under the terms and conditions of the Creative Commons Attribution license (http://creativecommons.org/licenses/by/4.0/).
